# Effect of radiation dose reduction on image quality in adult head CT with noise‐suppressing reconstruction system with a 256 slice MDCT

**DOI:** 10.1120/jacmp.v16i3.5360

**Published:** 2015-05-08

**Authors:** Ozdil Baskan, Cengiz Erol, Hanefi Ozbek, Yahya Paksoy

**Affiliations:** ^1^ Department of Radiology Istanbul Medipol University School of Medicine Istanbul Turkey; ^2^ Istanbul Medipol University School of Medicine Istanbul Turkey

**Keywords:** image quality, dose reduction, reconstruction methods, phantom studies, MDCT

## Abstract

The purpose of our study was to investigate the effect of iterative reconstruction (IR) as a dose reduction system on the image quality (IQ) of the adult head computed tomography (CT) at various low‐dose levels, and to identify ways of setting the amount of dose reduction. We performed two noncontrast low‐dose (LD) adult head CT protocols modified by lowering the tube current with IR which were decided in the light of a group of phantom studies. Two groups of patients, each 100 underwent noncontrast head CT with LD‐I and LD‐II, respectively. These groups were compared with 100 consecutive standard dose (STD) adult head CT protocol in terms of quantitative and qualitative IQ. The signal‐to‐noise ratio (SNR) of the white matter (WM) and gray matter (GM) and contrast‐to‐noise ratio (CNR) values in the LD groups were higher than the STD group. The differences were statistically significant. When the STD and the LD groups were compared qualitatively, no significant differences were found in overall quality. By selecting the appropriate level of IR 34%, radiation dose reduction in adult head CT can be achieved without compromising IQ.

PACS number: 87.57.‐s

## I. INTRODUCTION

Following its introduction by Godfrey Hounsfield in 1972,^1^, ^2^ CT has become an important method in medical diagnosis.^(3)^ The development of spiral CT — and thereafter multidetector CT (MDCT) — in the past few years has allowed a shorter examination time and thinner sections. CT use for diagnostic evaluation has increased dramatically all over the world.^4^, ^5^, ^6^, ^7^ The total number of CT examinations performed annually in the US has risen from 3.3 million in 1980–82 to 68.7 million in 2007.^4^, ^8^ As head CT has evolved as the technique of choice to assess traumatic and nontraumatic neurological conditions, its use has become particularly frequent.^(9)^ In the US, 19 million head CTs were performed (i.e., 28.4% of all CT procedures) in 2007.^(4)^

The most important problem with the increasing use of CT is the associated dose of ionizing radiation and the potential risk of cancer development later in life.^7^, ^10^, ^11^, ^12^, ^13^ To date, attention has focused on risks from pediatric CT scans. However, Berrington de González et al.^(13)^ estimate that, in terms of absolute numbers, the potential public health impact of current use patterns is highest for adults aged 35 to 54 years because of the high frequency of use. It is important for the radiologist to be sure that CT examination is required for the patient and that all efforts are made for reducing the radiation dose. Radiologists should have a control mechanism while reducing the dose in order not to sacrifice the image quality (IQ).

For that reason, many dose methods to reduce radiation exposure during CT examinations have been developed.^14^, ^15^, ^16^ Radiation dose reduction can be obtained by reducing tube potential (kVp) or current (mAs), resulting in increased image noise and decreased IQ.^14^, ^17^ With increasing computational power, a reconstruction method, statistical iterative reconstruction (IR), has become an alternative in overcoming these concerns while reducing radiation exposure.^(18)^ iDose^4^ (Phillips Healthcare, Best, the Netherlands) is one of the IR techniques can be used effectively, that was investigated in previous papers.^19^, ^20^

In this article, we study the effect of the radiation dose reduction on IQ in adult head CT with IR technique (iDose^4^) using a 256 slice MDCT. Because IQ decreases with the radiation dose, careful choice of the appropriate IR level is necessary. We try to describe a roadmap, while reducing the radiation dose, in order to achieve an optimal balance between image noise and quality, and compared our findings with the previous studies.

## II. MATERIALS AND METHODS

When we decided on the application of the IR in our department, we performed two noncontrast low‐dose (LD) adult head CT protocols with IR which were constituted in the light of a group of phantom studies. The local institutional review board approved this study. Because vendor experience and our prior phantom studies suggest preservation of diagnostic image quality at all dose levels used in our study, informed consent of patients was waived and all examinations were performed as standard of care.

All phantom and patient CT examinations were performed by using a 256 slice MDCT scanner (Brilliance iCT, Phillips Healthcare).

### A. Phantom studies

A female adult anthropomorphic phantom (model 702‐D; CIRS, Norfolk, VA), which contains tissue‐equivalent epoxy resins in all aspects of the phantom (the manufacturer supplied information, model 702‐D; CIRS) was used.

**Figure 1:**
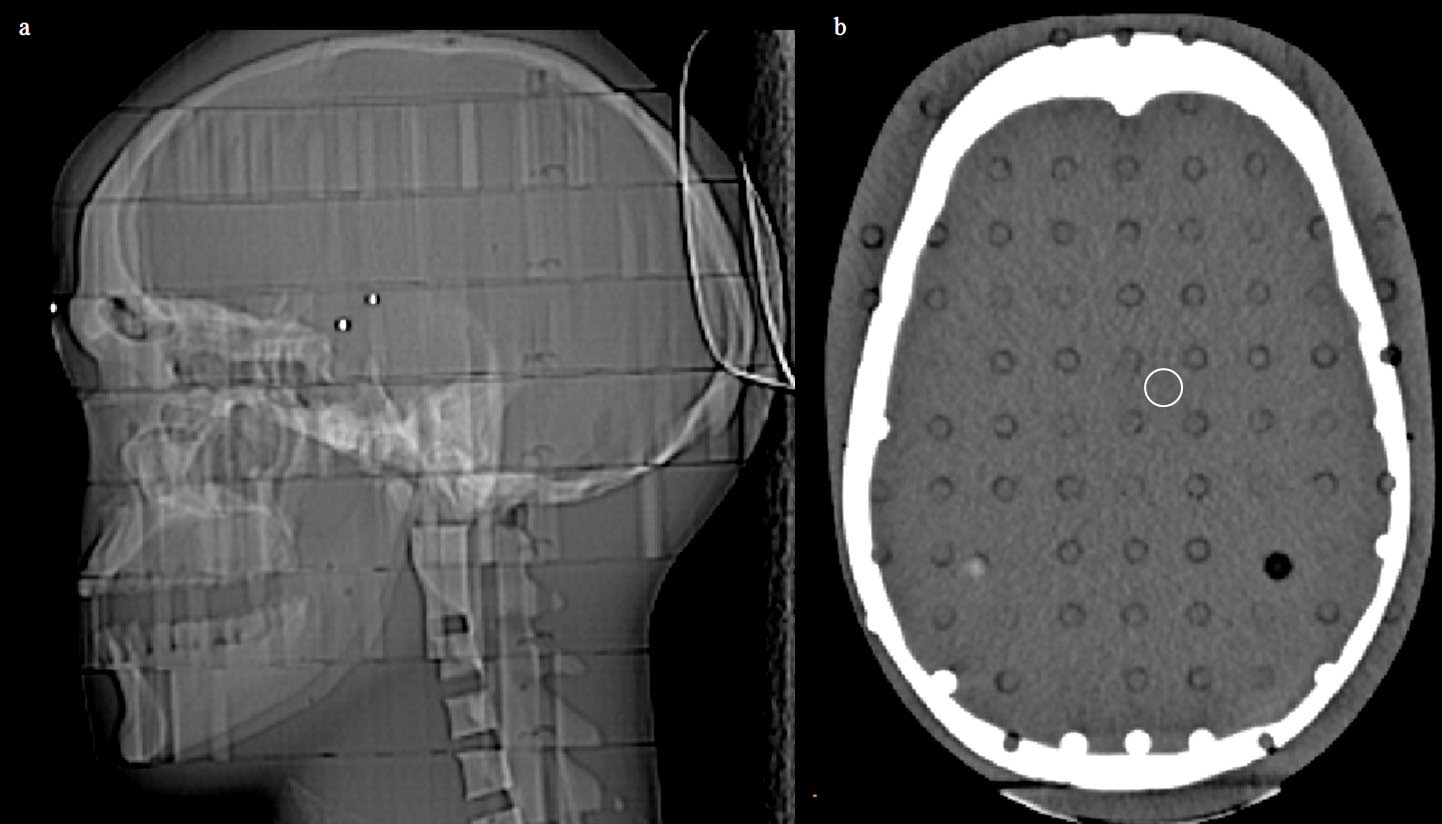
CIRS phantom: (a) lateral scout; (b) the axial slice scanned at 300 mAs, demonstrating region‐of‐interest placement.

The STD head CT protocol that is recommended by the vendor was the beginning point. The scan parameters are shown in Table 1. Subsequently, phantom scanning was repeated by reducing the mAs by 30 mAs, while all other scan parameters remained constant (300, 270, 240, 210, 180, 150). Each scan was reconstructed by the filtered backprojection (FBP) algorithm and five levels of the IR technique (iDose^4^ 1–5) that were predefined by the vendor. A total of 36 scans were analyzed. The noise (in SD) of each study was evaluated while 1 cm^2^ region of interest (ROI) was drawn in the same place (Fig. 1). According to our experience, multiple ROI measurements will lead to the more accurate conclusion. In this study, all (phantom and patient) ROI measurements were done in three consecutive slices and their average value was used. Additionally, we repeated the same protocol and method with 100 kV and 140 kV in order to check the reliability.

**Table 1:** The STD head CT protocol's scan parameters recommended by the vendor.

tube voltage	120‐kVp
tube current	300 mAs
pitch	0.391
Gantry rotation time	0.5 s
slice thickness and reconstruction interval	5 mm
detector collimation	64×0.625
field‐of‐view	25 cm

As a result of the noise information acquired through the anthropomorphic phantom measurements, Catphan 504 phantom (Phantom Laboratory, Salem, NY) was scanned with a STD and two LD head CT protocols. CT parameters were: STD protocol (as defined before) with mAs 300 and FBP reconstruction, LD‐I head CT protocol 250 mAs with iDose^4^2, LD‐II head CT protocol 200 mAs with iDose^4^3. All scanning parameters were identical, except mAs. These protocols were compared by using the four modules of the Catphan phantom for objective IQ before the patient applications.

The effect of dose and IR (iDose^4^) on image noise, SNR, CNR, uniformity, low‐contrast resolution, and high‐contrast resolution were evaluated. The methods and formulas were based on the literature^21^, ^22^, ^23^ and the information supplied by the manufacturer (Catphan 504 Manual, The Phantom Laboratory).

To measure the image noise, SNR and CNR linearity module (CTP 404) was used. Image noise was defined as SD of the 10 mm diameter ROI located in background at the center of the module.

SNR was determined by dividing the mean Hounsfield units (HU) value by the SD of the same ROIs.

(1) SNR=Mean HU of the ROI/SD of the ROI

CNR was determined by dividing the difference between the mean value obtained from the ROI in one of the round objects (acrylic) within the phantom and the mean value of the background by the SD of the background.

(2) CNR=Mean HU of the acrylic−Mean HU of background/(SD of the background)

The CT number (${\text{CT}}_{n}$) uniformity according to the location, edge, and center, was determined by using the image uniformity module (CTP 486). We measured the ${\text{CT}}_{n}$ (in HU) from the center of the module and at 3, 6, 9 and 12 o'clock locations of the module. All ${\text{CT}}_{n}$ for all five ROIs must be within ±5 HU of the center ROI mean value.

The low‐contrast module (CTP 515) was used for contrast detail evaluation. This module is made of several sets of cylindrical low contrast objects. We chose the 1.0% contrast level to determine whether the contrast detail changed visually and numerically. We evaluated the number and the diameter of the objects that are visible.

For the assessment of high‐contrast resolution, the high resolution module (CTP 528) was used.^(19)^ The high‐resolution bar patterns and the corresponding line pairs/cm ($\text{lp}/{\text{cm}}^{-1}$) were evaluated and the scans were compared.

### B. Patient groups

The first 100 patients underwent noncontrast LD‐I head CT protocol and the second 100 patients underwent LD‐II head CT protocol between October and December 2013. These groups were compared with 100 consecutive STD head CT protocol that we used before the application of the IR in our department during August to September 2013. No limitations were used for the indications. The CT scans (totally 18) with motion artifacts were excluded from the study.

### C. Radiation dose

The ${\text{CTDI}}_{\text{vol}}$ and DLP were obtained at the end of each scan from the dose information page of the scanner. ED in mSv was calculated by multiplying DLP (mGy*cm) with a constant adult head specific conversion factor (κ) 0.0021 mSv/ (mGy*cm).^(24)^

### D. CT data analysis

Head measurements were done for every patient from the CT images. We looked for the homogeneity between the patient groups. Anatomic landmarks were chosen. The distance between glabella and opisthocranion (GOD) and biparietal diameter (BPD) was measured.

### E. Quantitative image analysis

One radiologist who was not involved in the qualitative IQ analysis made quantitative measurements on CT images.

Image noise measurement was made by recording the SD of the circular ROI in the air, cerebrospinal fluid (CSF), bone, WM, and GM. SD and ${\text{CT}}_{n}$ were obtained for all series in all groups. The size, shape, and position of the ROIs were kept constant among the image sets for comparison by applying a copy and paste function at the workstation (Brilliance Workspace, Philips Healthcare). The ROIs of 1 cm^2^ in‐air were placed at the level of frontal sinus 1 cm away from the bone. The ROIs $\left(15-20\;{\text{mm}}^{2}\right)$ of CSF were drawn in the lateral ventricle corpus or the frontal horn. The ROIs $\left(50-60\;{\text{mm}}^{2}\right)$ for WM were placed in the frontal lobe and GM was placed in the thalamus. The ROIs $\left(25-30\;{\text{mm}}^{2}\right)$ for bone were placed in the clivus.

The SNR of the images were calculated for the WM and GM which were drawn in the frontal lobe and thalamus, respectively. SNR: ${\text{CT}}_{n}$/SD.

In order to calculate the CNR, four ROIs with 4 mm^2^ measurements in the GM and WM at the level of centrum semiovale of three consecutive slices were detected and get the averages of the ${\text{CT}}_{n}$ and SD values. The calculations were made by the following equation, as defined in the literature previously.^25^, ^26^, ^27^

(3) $$\text{CNR}:(mean\;GM\;HU-mean\;\text{WM}\;HU)/[(\text{SD}\;{\text{GM})}^{2}+(SD\;{{\text{WM})}^{2}]}^{1/2}$$

### F. Qualitative image analysis

All datasets were randomized and reviewed on the workstation (Brilliance Workspace, Philips Healthcare) for assessment of qualitative analysis. Two radiologists with more than 10 years of experience assessed all image datasets independently. They were blinded to the whole scan parameters. Images were graded for subjective noise, artifacts, and descriptiveness of the posterior fossa contents, GM–WM differentiation, and overall image quality on a five‐point Likert scale (1 worst to 5 best). For grading the descriptiveness of posterior fossa contents: visualization of the brain stem structures, contours of the basal cisterns and ventricular system, and cerebellar folia were evaluated. WM and GM differentiation: visualization and differentiation of the basal ganglia and the cortex were graded. We thought that these parameters reflect the lesion detectability of the images. Grades for image quality were averaged across both readers for further analysis.

### G. Statistical analysis

For the statistical analysis of the quantitative imaging parameters, one‐way ANOVA was used. A p<0.05 indicated statistical significance. The homogeneity of the variance of the study groups were analyzed by the Levene test. The groups that have p>0.05 were considered homogenous. Tukey's HSD test for homogenous groups and Tamhane's T2 test for nonhomogenous groups were used for post hoc analysis. The chi‐square test was used for gender comparison.

A nonparametric Kruskal‐Wallis variance analyze test and the Mann‐Whitney U test was used to determine statistical significance for the qualitative image analysis. Interobserver agreement in the assessment of image quality was quantified by weighted statistics (kappa statistics). Kappa value is a statistical measure of interobserver agreement for qualitative measurements. The κ value can be interpreted as 0.21–0.40 fair, 0.41–0.60 moderate, and 0.61–0.80 good.

## III. RESULTS

### A. Phantom study

CIRS phantom studies demonstrated noise reduction when the same tube current was reconstructed with increasing levels of iDose^4^ (Table 2). The noise levels at 20% and 30% decreased mAs reconstructed by iDose^4^2 and iDose^4^3, respectively, were almost equal to the STD with FBP algorithm. For that reason these iDose^4^ and mAs reduction levels were chosen for further study. The results were the same for the different tube voltages (100, 120, 140 k V, Table 2).

**Table 2:** The noise data (in SD) scanning of the CIRS head phantom at intervals of decreasing mAs and increasing iDose^4^ levels relative to STD head CT protocol using 120 kVp. The same protocol and method with 100 kVp and 140 kVp. $\text{mAs}=\text{tube current};\text{FBP}=\text{filtered backprojection};{\text{CTDI}}_{\text{vol}}=\text{volume CT dose index};\text{kVp}=\text{tube voltage};\text{LD}=\text{low dose};\text{STD}=\text{standard dose}$.

	*120 kVp*						
*mAs*	*FBP*	*iDose^4^1*	*iDose^4^2*	*iDose^4^3*	*iDose^4^4*	*iDose^4^5*	${\mathrm{CTDI}}_{\mathrm{vol}}$
300	3.3	2.9	2.8	2.6	2.3	2.1	41.24
270	3.7	3.3	3.2	2.9	2.7	2.5	37.1
240	3.8	3.5	3.2	2.9	2.7	2.4	32.96
210	4	3.6	3,4	3.2	2.9	2.6	28.99
180	4.4	3.9	3.6	3.4	3.1	2.8	24.67
150	4.5	4	3.7	3.4	3.2	2.9	20.53
	*100 kVp*						
300	4.4	3.9	3.6	3.4	3.1	2.7	25.18
270	5.1	4.5	4.3	3.9	3.6	3.2	22.65
240	5.5	4.8	4.5	4.2	3.8	3.4	20.12
210	5.5	4.9	4.6	4.4	3.9	3.4	17.59
180	6.1	5.5	5.2	4.8	4.5	4.1	15.07
150	6.9	6.1	5.7	5.3	4.8	4.4	12.54
	*140 kVp*						
300	2.6	2.5	2.2	2.1	1.9	1.7	60.17
270	2.9	2.6	2.4	2.2	2	1.8	54.13
240	3.1	2.8	2.6	2.4	2.2	2	48.09
210	3.5	3.2	3	2.7	2.5	2.3	42.3
180	3.8	3.4	3.2	3	2.7	2.5	36
150	4.1	3.7	3.4	3.2	2.9	2.6	29.96

When the STD, LD‐I and LD‐II protocols were compared by using the Catphan phantom, the background noise levels were lower, and the SNR and the CNR values were higher in LD protocols than the STD protocol (Table 3).

**Table 3:** The background noise levels, the SNR, and the CNR values of the STD, LD‐I and LD‐II protocols were compared by using the Catphan phantom, module 404. LD=low dose;STD=standard dose;CNR=contrast–to–noise ratio;SNR=signal–to–noise ratio.

	*STD*	*LD‐I*	*LD‐II*
background noise	3.9	3.5	3.7
background SNR	26.3	29	27.2
CNR	5.9	6.4	6.2

**Figure 2:**
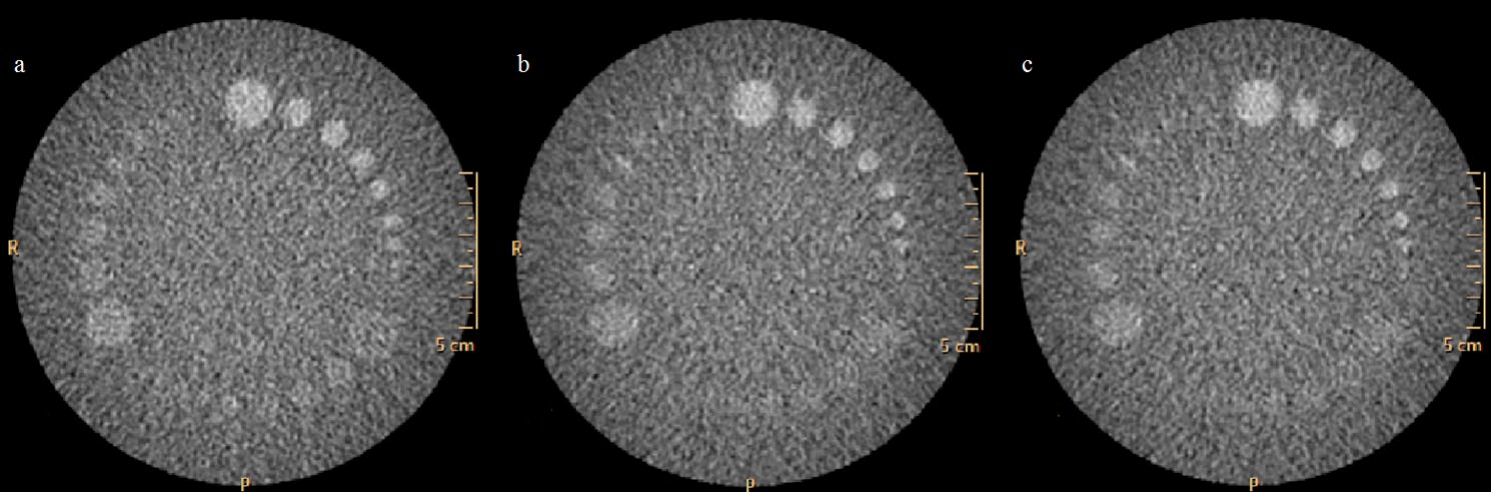
Catphan low‐contrast phantom module (CTP 515) is made of several sets of cylindrical low‐contrast objects: (a) STD protocol (300 mAs, with FBP reconstruction); (b) LD‐I (250 mAs, with iDose^4^2); (c) LD‐II (200 mAs, iDose^4^3). Comparison of low‐contrast images showed comparable visualization of the 7 disc objects, up to 4 mm diameter, at 1% in all of the protocols similar to data published in the literature. FBP=filtered backprojection;LD=low dose;STD=standard dose.

**Figure 3:**
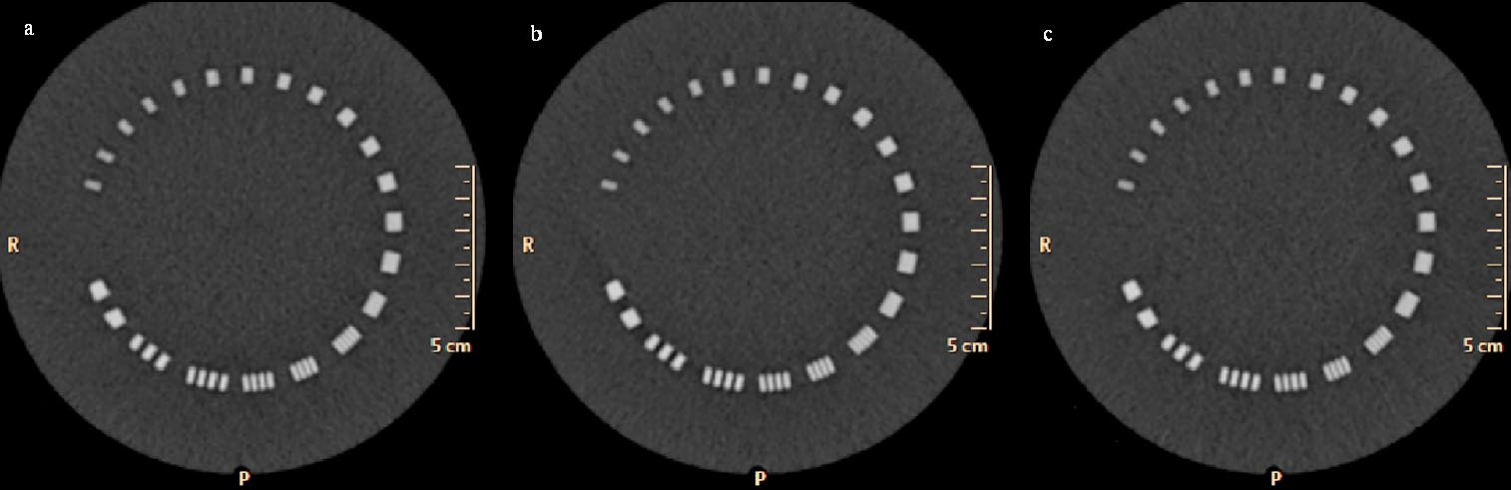
Catphan high‐resolution phantom module (CTP 528) with high‐resolution bar patterns: (a) STD protocol (300 mAs, with FBP reconstruction); (b) LD‐I (250 mAs, with iDose^4^2); (c) LD‐II (200 mAs, iDose^4^3). There were no differences in the identification of these high‐contrast resolution bar patterns between the groups. LD=low dose;FBP=filtered backprojection;STD=standard dose.

Field uniformity measurements were preserved, and the difference of the values according to the locations and the protocols were within the limitations, as described in the Materials & Methods section (Table 4). Comparison of low‐contrast images showed comparable appearances of the 4 mm objects at 1% in all of the protocols similar to data published in the literature (Fig. 2). There were no differences in the identification of these high‐contrast resolution bar patterns between the groups (5 lp/cm based on phantom guidelines) (Fig. 3). The low‐dose application with iDose^4^ did not decrease the high‐contrast resolution.

**Table 4:** Field uniformity measurements of Catphan phantom using CTP 486 module. ${\text{CT}}_{n}$ given in HU.LD=low dose;STD=standard dose;CTn=CT number;HU=Hounsfield units.

	*STD*	*LD‐I*	*LD‐II*
Center	17.2	17.56	17.56
3 o'clock	15.66	15.8	16.06
6 o'clock	16.03	15.6	15.56
9 o'clock	14.86	14.83	15.1
12 o'clock	15.83	15.76	15.7

### B. Patient demographics

There were no significant differences in the age and sex distribution between the groups (p>0.05) (Table 5). The BPD and GOD were similar in the groups (p>0.05) (Table 5). We achieved a significant dose reduction of 16.73% in ${\text{CTDI}}_{\text{vol}}$ and 16.06% in the DLP in LD‐I and 33.49% in the ${\text{CTDI}}_{\text{vol}}$ and 34.46% in the DLP in LD‐II group (p<0.0001) (Table 5). The ED (mSv) was significantly lower in both LD groups compared with the STD group.

**Table 5:** Patient characteristics and dose parameters of the three groups. LD=low dose;STD=standard dose;M=male;F=female;BPD=biparietal diameter;GOD=glabella and opisthocranion; ${\text{CTDI}}_{\text{vol}}=\text{volume CT dose index};\text{DLP}=\text{dose}–\text{length product};\text{ED}=\text{effective dose}$.

	*STD*	*LD‐I*	*LD‐II*	*p*
Age	46.6±19.4	45.2±20.3	44.4±17.46	0.7
Gender (M/F)	57/43	57/43	48/52	0.33
BPD	146.2±6.4	146.6±6.9	145.3±6.2	0.48
GOD	175±8.8	174.8±7.3	175.2±8.5	0.92
CTDIvol (mGy)	41.24	34.34	27.43	<0.0001
DLP (mGy.cm)	770±52	646±41	504±30	<0.0001
ED (mSv)	1.6±0.1	1.3±0.08	1.05±0.06	<0.0001

### C. Quantitative analysis

The noise levels of the CSF (STD‐LD‐I ρ=0.001, STD‐LD‐II p<0.0001), WM (STD‐LD‐I p<0.0001, STD‐LD‐II p<0.0001), and GM (STD‐LD‐I p<0.0001, STD‐LD‐II p<0.0001) in the low‐dose groups were significantly less than the STD group. The noise levels of the bone in the low‐dose groups were less than the STD group. The difference between the STD and LD‐II group was significant (p=0.032) and insignificant between STD‐LD‐I (p=0.603). The average noise value for air was high in LD‐II than the STD (p=0.030) and no difference is detected between STD and LD‐I groups (p=0.829) (Table 6).

**Table 6:** Mean and SD of noise, SNR and CNR in various CT protocols. (p‐values are mentioned in the main text). LD=low dose;STD=standard dose;CSF=cerebrospinal fluid;WM=white matter;GM=gray matter;SD=standard deviation.

	*STD*	*LD‐I*	*LD‐II*
NOISE			
Air	2.64±0.42	2.60±0.33	2.77±0.31
CSF	3.30±0.41	3.09±0.42	2.79±0.41
WM	2.94±0.38	2.71±0.32	2.75±0.34
GM	3.45±0.34	3.11±0.37	3.10±0.31
Bone	48.58±12.69	46.73±14.57	43.70±13.55
SNR WM	9.46±1.46	10.47±1.63	10.00±1.49
SNR GM	9.68±1.24	10.75±1.96	10.69±1.58
CNR	3.17±0.73	3.54±1.09	3.82±0.65

The SNR of the WM (STD‐ LD‐I p<0.0001, STD‐LDII p=0.033) and GM (STD‐ LD‐I p<0.0001, STD‐LDII p<0.0001) values and the CNR (STD‐ LD‐I p<0.0008, STD‐LDII p<0.0001) levels in the LD groups were higher than the STD group. The differences were statistically significant (Table 6). We did not observe any artifacts in either group that made interpretation impossible.

### D. Qualitative analysis

**Figure 4:**
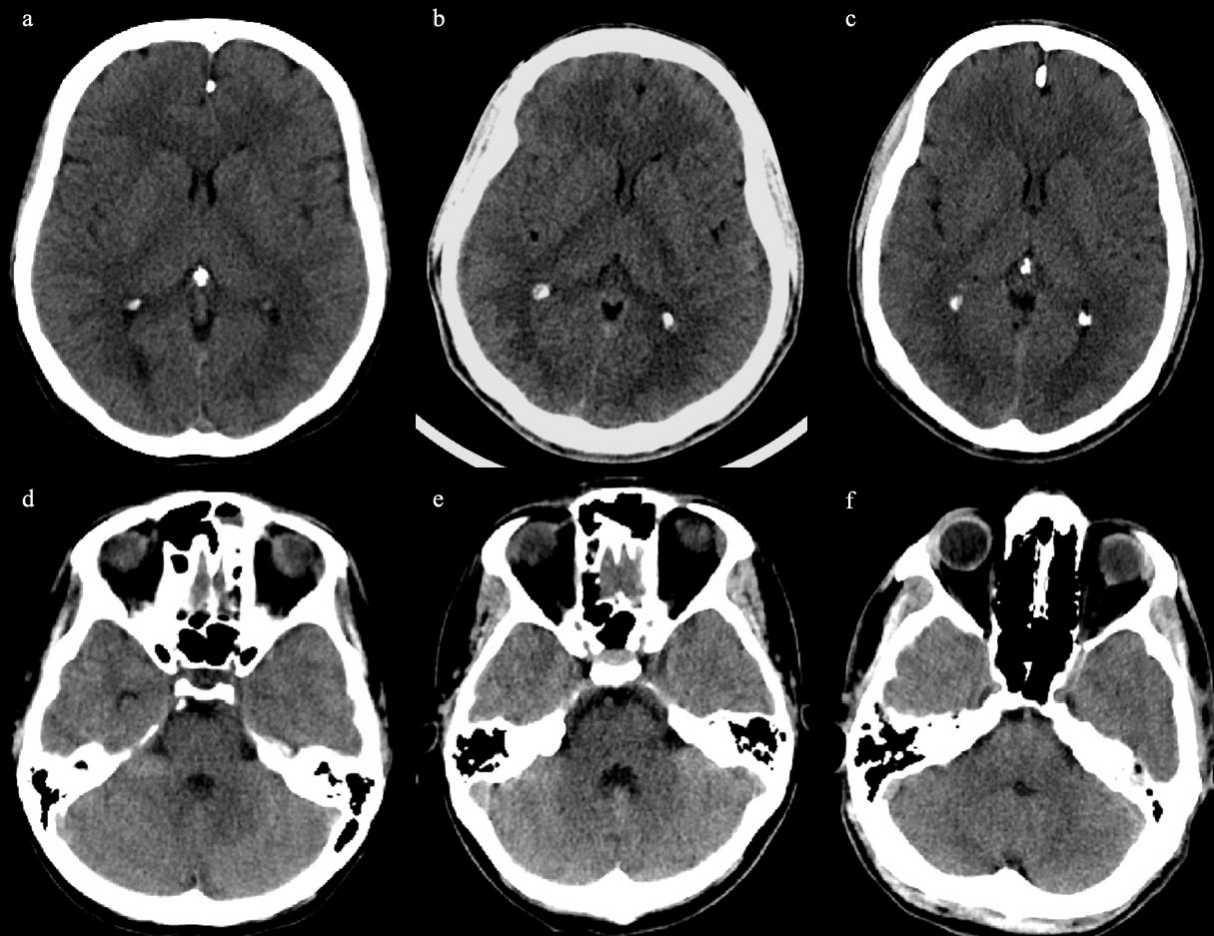
CT axial images of various protocols: (a) and (d) STD protocol (300 mAs, with FBP reconstruction); (b) and (e) LD‐I (250 mAs, with iDose^4^2); (c) and (f) LD‐II (200 mAs, iDose^4^3). The overall IQ is similar. FBP=filtered backprojection;IQ=image quality;LD=low dose;STD=standard dose.

When the STD and the LD‐I groups were compared, no significant differences were found in descriptiveness of the posterior fossa contents (p=0.225) and overall quality (p=0.083) (Fig. 4). The subjective noise (p=0.003), artifact (p=0.038), and GM–WM differentiation (p=0.045) scores were better in LD‐I group than the STD group. There were no significant difference in subjective noise (p=0.456), artifacts (p=0.697), and overall quality (p=0.306) between the STD group and LD‐II group. Scores for descriptiveness of the posterior fossa contents (p=0.027) and GM–WM differentiation (p=0.011) were better in the STD group than LD‐II group (Table 7).

**Table 7:** Mean and SD, median of qualitative image scores in various CT protocols (p‐values are mentioned in the main text). LD=low dose;STD=standard dose;SD=standard deviation.

	*STD*	*LD‐I*	*LD‐II*
Subjective noise	4.28±0.57(4)	4.45±0.55(4)	4.32±0.58(4)
Artifact	4.1±0.44(4)	4.19±0.42(4)	4.08±0.48(4)
Descriptiveness of posterior fossa	4.52±0.54(5)	4.59±0.51(5)	4.41±0.54(4)
GM–WM differentiation	4.22±0.62(4)	4.34±0.63(4)	4.07±0.6(4)
Overall image quality	4.19±0.46(4)	4.28±0.46(4)	4.14±0.47(4)

For the subjective analysis, the kappa values for interobserver agreement in evaluating various image quality parameters were moderate (weightedκ=0.56−0.61).

## IV. DISCUSSION

Due to the widespread use, MDCT has become the major source of radiation related to diagnostic imaging and this has raised concerns about radiation exposure in the general population. The risk of cancer from diagnostic X‐rays reported from 0.6% to 3.0% in developed countries.^(28)^

Radiation dose is proportional to the total number of the photons and the photon energy within the X‐ray beam.^(29)^ Many strategies have been introduced to reduce the radiation dose including optimizing the kVp and the mAs, automated tube current modulation, adaptive dose collimation, and noise‐reduction reconstruction algorithms.^16^, ^27^, ^29^ Unfortunately the basic methods, such as lowering the kVp or mAs, sacrifice the IQ.

Image noise and resolution are the major determinants of IQ. Reconstructed image noise is affected mostly by radiation dose and image‐processing methods.^(18)^ IR has focused on noise suppression and artifact reduction associated with lowering the radiation dose.^(29)^

In our study, a reduction of ${\text{CTDI}}_{\text{vol}}$ by 16.73% in LD‐I and by 33.49% in LD‐II protocols were achieved with no significant change in the overall image quality compared with the STD protocol. Similar findings were reported more recently. The application of various IR methods has been described to lower the radiation dose in head CT — in studies by Kilic et al.^(30)^ a 31% decrease in DLP, by Rapalino et al.^(31)^ a reduction of 26% in ${\text{CTDI}}_{\text{vol}}$, by Alper et al.^(32)^ a reduction of 38% in DLP achieved without affecting diagnostic acceptability. Another study published by Korn et al.^(33)^ compared their original nonenhanced and enhanced head CT FBP protocol with two LD IR protocols. The authors found that a 15% dose reduction did not contribute with IQ, but a 30% dose reduction resulted in degradation of IQ when reconstructed with IR (IRIS).

WM, GM, and CSF demonstrated significantly decreased noise levels in our LD protocols relative to the STD protocol. The CSF was the most prominent region. This is similar to the findings of the other studies which they attributed to the propensity of IR to reduce noise in smoother areas more prominently.^30^, ^34^, ^35^ Bone demonstrated significantly low noise in LD protocols which is not statistically significant in LD‐I. In previous studies no significant difference had been shown between the noise levels in WM and bone, which is not consistent with our study.^30^, ^34^ Additionally, we studied the GM noise and found lower levels in LD groups. Air noise measurements demonstrated no significant difference between the STD and LD‐I groups and was higher in LD‐II than the STD group. We think that this might be because of the small FOV in front of the frontal bone. We could not change the FOV in order to compare the LD and the STD groups.

The SNR of WM and GM and CNR in both LD groups were significantly higher than the STD group. Kilic et al.^(30)^ had demonstrated higher CNR in their LD and IR protocol; however, unlike our study, WM measurements (noise and SNR) and subjective scores of noise were better in the STD group. Rapalino et al.^(31)^ demonstrated that the use of IR (ASIR) improves measurements of SNR and CNR of GM and WM brain structures in reduced‐dose head CT scans for adult patients similar to our results.

The subjective imaging quality is dependent on the amount of noise and the effect of blurring while using IR with low radiation doses. The level of noise is inversely related to the tube potential,^(36)^ and the blurring effect is proportional to the level of IR used. They must be in equilibrium. If the level of IR is high, the images become blurred; if the level of IR is low, images will be noisy. Unlike other IR algorithms, (e.g., ASIR; GE Healthcare, Waukesha, WI), the exact percentage of IR in different levels of iDose^4^ is not clearly demonstrated in the CT protocol. In order to avoid excessively noisy or smooth image reconstructions,^31^, ^35^ the recommended levels by the vendor might be followed.

In our study, we tried to find out the optimum IR level by performing a group of phantom studies before the patient applications which is described in the literature.^34^, ^37^ We evaluate the low‐contrast and high‐resolution quality of the protocols which are especially important for head and neck protocols.^(19)^ There were no differences between the protocols. The SNR and CNR became higher by the iDose^4^ levels in lower doses.

We achieved subjective differences in sharpness by evaluating the GM–WM differentiation and the posterior fossa descriptiveness. They were found to be better in the STD group than the LD‐II in qualitative evaluation, while the SNR and CNR were better in quantitative and phantom evaluations. Kilic et al.^(30)^ observed better image sharpness scores in the STD group than in the LD group. The authors attributed this to the oversmoothing effect of IR (ASIR) as well as to the noise. Wu et al.^(38)^ has mentioned that the blurring of imaging texture became evident in the infratentorial region. When the IR is increased unproportionately, a noiseless appearance occurs in the images, and radiologists may evaluate these images as oversmoothed or artificial, neither of which are desired.^(31)^ Changing the algorithm of the routine procedures from FBP to IR might be difficult. Perhaps for that reason, while the phantom and quantitative evaluation of the LD‐II protocol CNR and SNR values were better and comparable with the STD protocol, the scores of the qualitative evaluation were lower. The interobserver agreement for qualitative measurements (kappa values) was lower in LD‐II scores, too.

Differences in image sharpness might also depend on physical features of the patient. Variations in cranial thickness and amount of subcutaneous tissue surrounding the calvarium may contribute to subjective image sharpness.^(32)^ For that reason we measured the BPD and GOD and compared them as variables. Wu et al.^(38)^ had found that the head diameter was the only significant factor inversely correlated with infratentorial imaging quality. Head diameter cranial bone structures and thickness of the scalp might be investigated as variables while making the dose regulations and deciding on the amount of the radiation dosage. One of the limitations of our study is that we measured the diameters between the outer tabulas and didn't consider the amount of subcutaneous tissue in the calvarium. The thickness of the supratentorial and infratentorial diploe might be measured separately and compared among the groups as variables. This can be studied and compared, and some standardization might be found such as body mass index.

The posterior fossa was the part most affected by the lowering of the dose. We evaluated the posterior fossa qualitatively, but did not evaluate it quantitatively. This is the one of the limitations of our study. Artifacts, such as the operation materials, might be studied more specifically. We did not evaluate the special pathologies and the diagnostic accuracy of the protocols, which is another limitation of our study.

We additionally repeated the same procedure with 100 kVp and 140 kVp instead of the 120 kVp of our STD protocol. Our phantom data suggest that additional radiation dose reduction may be feasible with similar image noise, if the studies are reconstructed with a higher level of IR (e.g., 120 kV 180 mAs iDose^4^4) or by changing kV (e.g., 100 kV 300 mAs iDose^4^3, 140 kV 150 mAs iDose^4^2). These protocols can be used in future studies. We used only the noise level, but the CNR can be added too.

## V. CONCLUSIONS

Radiologists and physicists should be familiar with such dose‐reduction techniques and should optimize the imaging protocols to achieve the best images with a lower radiation dose. For analyzing various protocols, the vendor's phantoms might be used. The phantom studies can be used as a preview before trying a new dose‐reducing method. Our study showed that, by selecting the appropriate level of IR, radiation dose reduction up to 34% can be achieved in adult head CT examinations without compromising the overall IQ.
